# Traceability of Blood Transfusions and Reporting of Adverse Reactions in Developing Countries: A Six-Year Postpilot Phase Experience in Burkina Faso

**DOI:** 10.1155/2018/7938130

**Published:** 2018-12-20

**Authors:** Salam Sawadogo, Koumpingnin Nebie, Tieba Millogo, Sonia Sontie, Ashmed Nana, Honorine Dahourou, Dieudonné Yentema Yonli, Jean-Baptiste Tapko, Jean-Claude Faber, Eléonore Kafando, Véronique Deneys

**Affiliations:** ^1^University Ouaga I Professor Joseph KI-ZERBO, 03 BP 7021 Ouagadougou 03, Burkina Faso; ^2^National Blood Transfusion Centre, 01 BP 5372 Ouagadougou, Burkina Faso; ^3^African Institute of Public Health, 12 BP 199 Ouagadougou, Burkina Faso; ^4^African Society for Blood Transfusion, Cameroon; ^5^Association Luxembourgeoise des Hémophiles, 33 rue Albert Ier, 1117, Luxembourg; ^6^CHU UCL Namur asbl, 15 place L. Godin, 5000 Namur, Belgium

## Abstract

Traceability is an essential tool for haemovigilance and transfusion safety. In Burkina Faso, the implementation of haemovigilance has been achieved as part of a pilot project from 2005 to 2009. Our study aims to evaluate the traceability of blood transfusions and reporting of adverse reactions over the 6-year postpilot phase. A cross-sectional study including all blood units ordered between 2010 and 2015 has been conducted in public and private health care facilities supplied with blood products by the transfusion center of Bobo-Dioulasso. The complete traceability was possible for 83.5% of blood units delivered. Adverse reactions were reported in 107 cases representing 2.1/1,000 blood units per annum. Transfusions of wrong blood to wrong patient were reported in 13 cases. Our study shows that the haemovigilance system in Burkina Faso must be improved. Healthcare workers have to be sensitized on how traceability and haemovigilance could impact the quality of care provided to patients.

## 1. Introduction

Blood transfusion is a life-saving treatment, generally used to replace blood lost in surgery and obstetric or to treat life-threatening anemia and inherited blood disorders. However, it is an event which carries potential risks of acute and/or delayed transfusion reactions and transfusion-transmitted infections for the recipient [1]. Therefore, it is necessary for blood services and clinical services to control the entire process through an effective Quality Management System (QMS) in order to reduce these risks.

Blood transfusion process comprises a series of steps including among others ordering of blood or blood products, administration of blood, monitoring of the transfused patient, managing of adverse reactions, and documentation of transfusion adverse events and outcomes [2].

In transfusion practices, the traceability of blood products means that, at any time, blood transfusion services must know “*who donated or who received which blood or blood product*?” Therefore, their QMS must have documentation systems for information allowing following a blood product or the procedure from the donor to the recipient (vein-to-vein) and vice versa. This implies a close collaboration between blood services and clinical services. The principle was gradually established since the scandal of HIV-infected blood in the late 1980s. The basis for this implementation is the possibility of ascending and descending surveys or look-back studies, which form undoubtedly the basis for improvement and optimization of transfusion safety [3]. Thus, traceability of blood products is an essential tool for haemovigilance and transfusion safety. Its objective is to retrieve from a donation number, the history of the donor and the recipients of the blood products processed from the donated blood [4].

This is possible if a system of information shared between blood transfusion services and health care units is in place and well functioning. In Europe, haemovigilance systems are well-defined, in which the responsibilities of each institution and every stakeholder are well described [5]. The annual report 2015 of the Haemovigilance Authority in France indicates that the average national traceability rate was 99.2% [6]. In Morocco, the traceability rates were around 51% in Casablanca [7] and 15.5% in Rabat [8].

In Burkina Faso, the National Blood Transfusion Center (CNTS) has put in place a QMS according to the ISO 9001 standards. In documents including a blood policy, quality manual and standard operating procedures (SOPs) on blood collection, blood products processing, blood products storage, and distribution, haemovigilance has been put in place. On the other hand, in many hospitals in the country, the QMS is very embryonic. There are almost no SOPs for clinical use of blood. The implementation of the haemovigilance system has been conducted as part of a pilot project from 2005 to 2009. The first data published in 2012 showed that the traceability rate was around 91% [9]. This project, supported by the World Health Organization (WHO), comprised (1°) health staff training and supervision (around 200 employees trained), (2°) design and dissemination of materials for transfusion traceability and adverse reaction reporting, and (3°) implementation of hospital transfusion committees [9].

It is well known that, in sub-Saharan African countries, one of the major challenges during the postproject phase is the sustainability of the achievements. Therefore it was important to raise the question of what happened after the end of the project of haemovigilance was implemented in Bobo-Dioulasso. Our study aimed at evaluating the traceability of blood transfusion and reporting of adverse reactions/events related to blood transfusion in public and private health care facilities supplied with blood product by the Regional Blood Transfusion Center of Bobo-Dioulasso.

## 2. Material and Methods

### 2.1. Study Setting and Design

A 6-year (2010-2015) retrospective evaluative study on haemovigilance in clinical transfusion was conducted in some health care facilities dependent on Regional Blood Transfusion Center of Bobo-Dioulasso (CRTS-B), one of the four regional blood centers in Burkina Faso. These facilities are comprised of the teaching hospital SANOU Souro of Bobo-Dioulasso, the 3 district hospitals of Dafra, Do, and Dandé, and a dozen relatively small private health care facilities located in Bobo-Dioulasso which are supplied with blood products by the CRTS-B. Clinicians from these care facilities receive the needed transfusion advice from the CRTS-B and report to this center the adverse events they observe during blood transfusion.

CRTS-B is one of the operational structures of the CNTS located in the western part of the country. The QMS implemented takes the CRTS-B into account. Indeed the entire system (organization and documentation) is harmonized at the national level for all the blood services affiliated to CNTS.

Blood is collected from voluntary and nonremunerated donors at fixed site and mobile collection. It is systematically screened for the following transfusion transmissible infections (TTIs): HIV, hepatitis B and C, and syphilis. The blood transfusion system policy is to process whole blood into blood products. Whole blood is stored in controlled cold rooms and processed, within 96 hours, into packed red blood cells (RBCs) mainly, over 90-95%, with regard to epidemiological profile and clinicians' needs. Being given the demand of therapeutic plasma and platelets is very low, as previously stated [9], a few number of whole blood units (5-10%), mainly from fixed collect sites, is processed into frozen fresh plasma (FFP) and platelet concentrates (PCs), within 6 hours after collection. Nontherapeutic plasma produced from RBCs processing is discarded.

Blood products are ordered by physicians on a standardized blood ordering form and delivered free of charge to patient. Delivered blood units are packed in cool-boxes and transported within 30 minutes to the clinical wards by employees of these services. The most distant service is located 20 minutes from blood delivery point. Each product delivered is accompanied with a posttransfusion and haemovigilance form (FPTH) on which the clinician has to record summarized information on administration of the blood unit and occurrence of adverse reactions. This form must be sent back without delay (as soon as possible) after blood transfusion, to the CRTS-B as transfusion confirmation and adverse reaction report, if applicable. The adverse reactions/events reporting system in place is a nonmandatory one.

### 2.2. Patients and Methods

Patients of both sexes and all ages admitted in private and public (teaching and district) hospitals, for whom blood was ordered between 2010 and 2015, were included in the study. Both medical and transfusion process information of each patient were recorded on medical software (CTS server). We extracted from this software the following information: age and gender of the recipient, hospital and ward of admission, date of blood delivery, type and number of blood products requested and delivered, confirmation that blood product was transfused, adverse events, and reactions reported where applicable.

### 2.3. Statistical Analysis

Excel (Microsoft Office 2007) and STATA 13 software were used for data management and data analysis. We used proportion and mean ± 2 SD to describe, respectively, qualitative and continuous variables. We used median and interquartiles 25 and 75% to describe the age of patients. The number of adverse reactions is divided by the number of traceable blood units and multiplied by 1,000 to have the incidence of adverse reaction per 1,000 blood units. The Chi-square (*χ*2) test was used to test the differences in frequencies between groups. Groups were assumed to differ significantly when the p-values were less than 0.05.

### 2.4. Ethical Considerations

The study was conducted with the approval of the directorate in charge of scientific and medical activities of the National Blood Transfusion Center (CNTS). Data were collected anonymously. Patient and donor confidentiality were preserved.

## 3. Results

From 2010 to 2015, 61,678 blood units including 59,934 RBC units were delivered to 42,269 patients (i.e., 1.5 blood units per patient and 10,280 units per annum). The median age of the patients was 13 years (IQR [2  -  30]). The majority of patients (59.5%) were female (the sex ratio M/F was 0.68). The female patients received 62.9% of blood units delivered (Chi2=78.5; p < 0.001).  Table 1 describes the distribution of patients, blood units ordered, blood units delivered and satisfaction rate per year, and type of hospital, of clinical ward, and of blood products.

**Figure 1:**
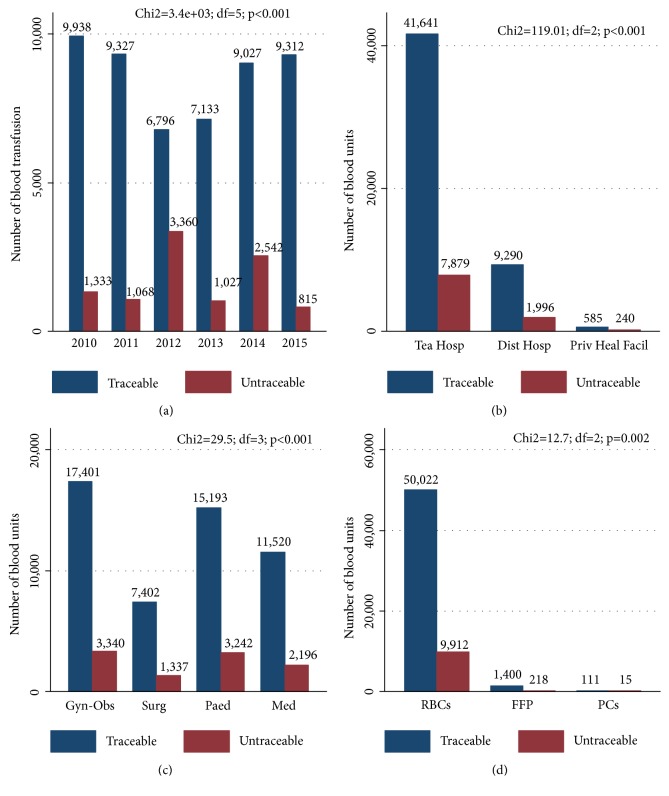
Number of blood units plotted compared to number of untraceable blood units per (a) year of delivery, (b) type of hospital, (c) clinical ward, and (d) type of blood products at the Regional Blood Transfusion Center of Bobo-Dioulasso from 2010 to 2015. Tea Hosp = Teaching Hospital; Dist Hospt = District Hospital; Priv Heal Facil = Private Health Facilities; Gyn-Obs = Gynaecology-Obstetrics; Surg = Surgery; Paed = Paediatrics; Med = Medicine; RBCs = Red blood cells; FFP = Frozen fresh plasma; PCs = Platelet concentrates.

Out of the 61,678 blood units delivered, the complete traceability of blood unit (“who received the blood, when, where, and from whom?”) was possible in 51,533 cases (83.5% of blood units delivered). A total of 50,033 FPTH were received (i.e., 97.1%) concerning the RBC units delivered. In private health facilities, feedback was sent for only about 29% of blood units delivered. This situation concerned 16% of the products delivered in public hospitals.  Figure 1 summarizes the number of blood units plotted compared to untraceable blood units according to (a) year of delivery, (b) type of hospitals, (c) clinical ward, and (d) type of blood products.

**Figure 2:**
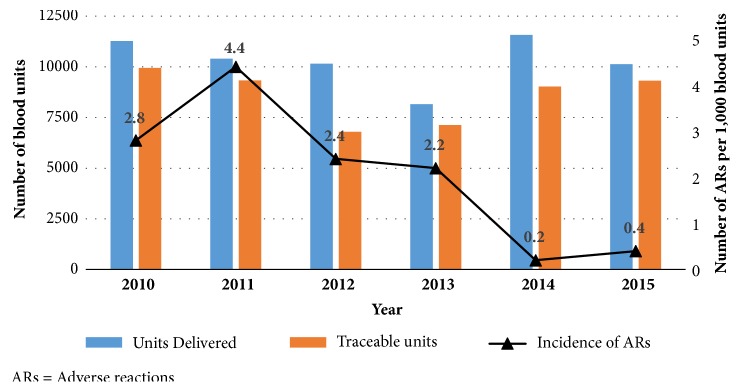
Incidence of adverse reactions reported to the Regional Blood Transfusion Center of Bobo-Dioulasso from 2010 to 2015, Burkina Faso.

**Figure 3:**
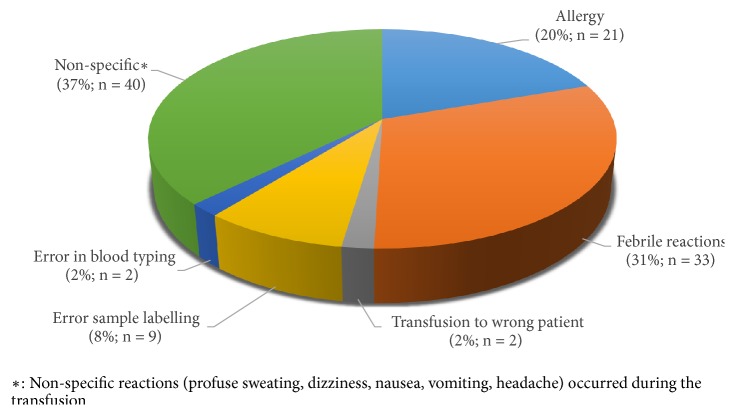
Types of adverse reactions reported to the Regional Blood Transfusion Center of Bobo-Dioulasso from 2010 to 2015, Burkina Faso.

A total of 107 adverse reactions were reported over the six years. This represented 2.1 reactions per 1,000 blood units per annum, taking into account the fact that the feedback rate (proportion of FPTH returned) was 83.6%.  Figure 2 shows the incidence of adverse reactions from 2010 to 2015. The reactions were nonspecific in 40 cases (i.e., 37%). They include symptoms such as sweating, dizziness, nausea, vomiting, and headache occurring during the transfusion.  Figure 3 gives the frequency and type of the adverse reactions reported. Incorrect blood component transfusions (IBCT) were reported in 13 cases (i.e., 0.3 cases per 1,000 units). In 2 cases, the blood unit was transfused to a patient different from whom it has been issued for. The other cases were due to errors in blood sample labelling (9 cases) and blood group typing (2 cases).

Besides these adverse reactions, death of the patient occurred during the blood transfusion in 65 cases (1.3 per 1,000 units). But since no investigation was conducted on these deaths, it was not possible to incriminate or not blood products. In addition, 53 units of RBCs (1.06/1,000) delivered to clinical services were returned to the blood transfusion service due to blood clots present in the unit.

## 4. Discussion

Haemovigilance is an important tool for improving blood transfusion safety. Indeed, the WHO global strategy for safe blood transfusion defined haemovigilance as one of its important pillars [10, 11]. It is recommended to each country to build an effective haemovigilance system. The objective of such a system is to report and analyze the adverse events and reactions related to blood use in order to implement measures to correct and prevent them.

This study is one of the few studies in sub-Saharan Africa, although the need of science-based evidence to improve transfusion safety in Africa is crucial. This is the second of its kind that was carried out in our country. It revealed that the system implemented at the Regional Blood Transfusion Center of Bobo-Dioulasso allowed the traceability of 83.5% of blood units delivered and reported 2.1 adverse reactions per 1,000 units per annum.

The implementation of the haemovigilance system at the CRTS of Bobo-Dioulasso and later throughout the country has been a long and inclusive process that had combined information, training and regulation as described by Dahourou et al. in a previous study [9]. The system is theoretically well-structured with a national committee, regional committees linked to regional health directions, and hospital transfusion committees [12]. It is a variant of the French model that was implemented in Burkina Faso. Nevertheless, the blood units' traceability rate in our study was 8% lower than in the pilot phase (83% versus 91%). These findings show that the habits and good practices acquired by healthcare workers during this pilot phase tend to be lost less than a year after, even if the results remain better than those in other African countries. This suggests the need of more regulation, continuous awareness-raising action and training, effective supervision, and control of stakeholders and health facilities through regular audits and effective communication system. As described by Dahourou et al., the pilot phase of the implementation of the haemovigilance system was marked by proactive attitude of the Regional Blood Transfusion Center which organized regular training and awareness sessions for clinicians and feedback on haemovigilance indicators for each hospital [9]. It is necessary to motivate the healthcare workers, for whom transfusing blood is far from being the only concern. Indeed, traceability of blood transfusions is sometimes perceived as an administrative constraint, more than a tool to improve transfusion safety [8]. Thus, the national and regional vigilance committees and the hospital transfusion committees are invited to fully assume their role. The nonmandatory and passive reporting system could also be questioned. Despite the existence of national guidelines for good practice in blood transfusion [13], there is no legal way to oblige clinical teams to comply with all the rules. As a result, CNTS, on its own initiative, has put in place measures to require from clinicians, feedback of the first units issued for the patients in their ward before receiving additional ones, while ensuring no occurrence of any delay in blood issuing procedure that might be harmful to critical patients. These “coercive measures” are coupled with an active approach such as information, training and supervision of hospital staff involved in transfusion activities [14]. This allowed substantial progress in haemovigilance in our country [15]. But most of the time, FPTH are returned only when another blood unit is requested, often several days or weeks after the last transfusion. In such cases, it was often impossible to investigate and to determine the relation with blood transfusion. So, this shows clearly that the measures implemented by the CNTS cannot replace a strong oversight by a regulatory authority (independently of blood services and care units). The regulatory authority, planned in the national blood policy, has yet to be put in place [12, 16]. For few years by now, the CNTS is advocating the Ministry of Health to implement this independent regulatory authority.

Traceability rates in our study were higher as compared to those found in Morocco (15.5% in Rabat in 2010 and 51% in Casablanca in 2003) [7, 8]. But it was lower than in developed countries like France [6]. Haemovigilance systems in developed countries have been established since a long time (often 1994-1996), as a reaction to the human immunodeficiency virus scandal in the late 1980s/early 1990s [17]. In Africa, implementation of the first haemovigilance systems started later [9, 18].

Adverse reactions ratio was 7 times lower in our study than in the pilot phase (2.1 versus 16.1 cases per 1,000 units). In Morocco and Tunisia, the ratio varied from 0.5 to 1.6/1,000 [7, 19] and from 0.59 to 2.19/1,000 blood units [20], respectively. In Namibian, Zimbabwean, and South African systems, these ratios were, respectively, 0.8, 0.46, and 0.82 per 1,000 blood units [18, 21, 22]. In a randomised controlled trial and prospective observational studies, JP Allain et al. [23] and AK Owusu-Ofori et al. [24] reported an adverse reactions ratio 5 to 10 times higher than our findings. The main differences with findings in these different countries could be explained by the system of adverse reactions' monitoring. In Ghana the adverse events have been noted during observational studies, while in other countries they were routine notifications; the context of the study probably affected the reporting of adverse events in Ghana.

In our study, the reported adverse reactions showed clinical symptoms that occurred during the blood transfusion or immediately after. Indeed, operational procedures and guidelines for clinical blood transfusion recommend monitoring of patients during and after blood administration, through the measurement of vital signs such as temperature, pulse, and blood pressure. But this is not well-codified and well-respected because of the insufficiency in staff number and more likely the lack of knowledge in importance of this monitoring on the quality of care and safety for patient. Adverse reactions documented on FPTH included mainly febrile reactions, chills, pruritus, profuse sweating, dizziness, etc. But many cases were reported to blood services several days or weeks posttransfusion, making any further rigorous investigation impossible. So, the severity of most of these reactions and the imputability on the transfused blood products could not be assessed. Only a few cases resulted in investigations leading to the detection of incorrect blood products transfused (wrong blood transfusions) due to patient misidentification, sample labelling and blood group typing errors. The incidence of IBCT was 0.3 cases per 1,000 units. These data are 1.5 times lower than previously observed in our country [9] and 10 to 100 times higher than those reported in Morocco (0.0025 to 0.02 per 1,000 units) and South Africa (0.03/1,000 units) [7, 19, 22]. The incidence was approximately the same as in Tunisia (0.24/1,000 units) [20].

The incorrect blood component transfusions (IBCT) represent a universally recognized cause of posttransfusion morbidity and mortality. Data in the literature show that, in around half of the cases, multiple errors occurred in the process and in more than 2/3 of the cases, the errors occurred in clinical wards. The most frequent errors are failure to perform correctly the pretransfusion controls (e.g., patient bedside test to ensure that the right blood is given to the right patient) [25, 26]. Indeed, the verification of identity concordance between the patient, the transfusion documents, and the blood product intended for transfusion at the patient's bedside are tainted by bad practice and misinterpretation [27]. In 11 cases out of 13 (i.e., 85%) in our study, errors in sample labelling and documentary verification were incriminated.

In France [6], with around 3.2 million blood units delivered in 2015, the incidence of adverse reactions was 2.4/1,000 units and the incidence of IBCT was 0.004 / 1,000 units, i.e., 100 times lower than our findings. This difference could be explained by the long culture and rich experience of haemovigilance in France.

Febrile reactions (33% of all adverse reactions) reported in this study, with regard to their clinical evolutions, were concordant with febrile nonhemolytic transfusion reactions (FNHTR). The FNHTR is a common adverse reaction occurring during blood transfusion. It could be related to the presence of cytokines in blood products, but in many cases, the mechanism remains uncertain [1, 28]. The other adverse reactions (37% of all cases) that were found in our study were nonspecific and included anxiety, vomiting, nausea, headache, and profuse sweating. These were benign symptoms presented by patients during the blood transfusion. In 65 cases (i.e., 1.3/1,000 blood units), only the mention “death during transfusion” was reported on FPTH. But there was no evidence for imputability on the transfusion of the patients' death, since no investigation was conducted. This kind of adverse reaction seems to be rather frequent and we need to put in more effort to investigate them. According to the literature, the transfusion-related acute lung injury (TRALI) and the acute haemolytic transfusion reactions are the main causes of transfusion-related deaths [27]. In our context, many patients arrive to health facilities very late and in very poor condition. This could explain our findings.

The blood clots detected in each one thousandth blood unit issued (1.06/1,000 units) could certainly be considered as grade 0 adverse events, but they constitute a serious concern. It reflects either deterioration in the quality of anticoagulant contained in collection bag or abnormalities during the collection (poor mixing of the blood with the anticoagulant). Anyway, the lack of or the poor implementation of visual inspection procedures of blood units during the processing process and mainly the delivery process is obvious.

Our study reported only data about acute adverse reactions. The FPTH, the notification materials in our system, were designed specifically for this purpose. Indeed, the primary goals of this form are the traceability of blood transfusions and the reporting of acute reactions. This means that, after the transfusion, the form must be returned immediately to the blood transfusion services. The primary aim was to create a reporting habit with the clinicians. If any adverse reaction occurs and the FPTH is returned within a reasonable period of time, the transfusion service in collaboration with the clinical department can investigate, grade the severity of reactions, and assess the imputability of blood products. In this case, a second form named “transfusion incident form (FIT)” is used. But, it is obvious that this process in not complete for all reported incidents. Only a few cases have been investigated.

Our findings show that the Regional Blood Transfusion Center of Bobo-Dioulasso does not cover the needs in blood products of the health facilities. The average satisfaction rate of blood demands was 90.2% between 2010 and 2015. This is due to insufficient blood collection, despite the many efforts made to improve the availability of blood over the last two decades (7 units donated per 1,000 inhabitants) [29]. The high rate of unmet blood demands for patients in obstetric and surgical wards (respectively, 10.5% and 22.6%) is a big concern, given that sometimes, they are in hemorrhaging and severely anemia situations that can compromise their survival. In 2012, the unmet blood requests for patients admitted in maternity were 15.6% in Ouagadougou [30].

In addition, the satisfaction rate for PCs requests was very low (22.6% compared to 90.7% and 94.3%, respectively, for RBCs and FFP). This indicates that the organization put in place for processing PCs seems to be ineffective. The platelet-rich plasma's method is used to process platelet concentrates. In a previous paper [31], we mentioned the poor setting of blood transfusion services in Burkina Faso (limited financial and material resources, shortage of trained staff, etc.), obliging them to use alternative techniques and strategies in order to make available, with a certain level of quality, the most requested blood products in hospitals. In our context, the main indications for blood transfusion are obstetrical hemorrhage and anemia caused by malaria, malnutrition, and other genetic diseases (sickle cells disease, thalassemia, etc.). This could explain the high proportion of RBCs units ordered.

Limitations to our study were that we did not report data on delayed adverse reactions and near-miss events, being given they are known to be the occult part of the errors occurring in blood transfusion. Besides that, the high number of undefined adverse reactions and patients dead during transfusion hides many abnormalities and nonconformities for which our system must put in more efforts to investigate and identify in order to improve transfusion safety.

## 5. Conclusion

The analysis of our haemovigilance system shows that our results, while being better than those in some other African countries, remain insufficient and decline from one year to the next, since the end of the pilot phase. This finding calls for the implementation of a quality system and management integrating haemovigilance in hospitals and indicates the need to strengthen the system in transfusion services.

Feedback and adverse reactions reporting in our system still use paper documents. Without any effective active approach, filling these papers is perceived as additional work by health staff. Implementing an electronic system could improve traceability and reporting rates, secure collection of data, and facilitate their exploitation. Anyway, corrective actions through training and regular awareness-raising of healthcare workers on how a strong traceability and haemovigilance system integrated to a quality assurance programme can improve the safety and effectiveness of care provided to patients have to be implemented.

## Figures and Tables

**Table 1:** Distribution of patients, blood units ordered, blood units delivered and satisfaction rate per to year, type of hospital, clinical ward, and type of blood products at the Regional Blood Transfusion Center of Bobo-Dioulasso from 2010 to 2015.

	**Patients (**%**)**	**Blood units ordered (**%**)**	**Blood units delivered (**%**)**	%** satisfaction of blood demand**
**Overall**	42,269 (100)	68,381 (100)	61,678 (100)	90.2%
**Years**				
2010	6,430 (15.2)	12,384 (18.1)	11,271 (18.3)	91.0%
2011	6,317 (14.9)	11,297 (16.5)	10,395 (16.8)	92.0%
2012	6,984 (16.5)	11,079 (16.2)	10,156 (16.5)	91.7%
2013	5,950 (14.1)	9,252 (13.5)	8,160 (13.2)	88.2%
2014	8,508 (20.1)	12,545 (18.3)	11,569 (18.7)	92.2%
2015	8,080 (19.1)	11,824 (17.3)	10,127 (16.4)	85.6%
**Hospitals**				
Teaching hospital	33,678 (79.7)	52,548 (76.8)	49,520 (80.3)	94.2%
District hospitals	8,017 (19.0)	14,687 (21.5)	11,286 (18.3)	76.8%
Private health facilities	533 (1.3)	1,091 (1.6)	825 (1.3)	75.6%
Not recorded	41 (0.1)	55 (0.1)	47 (0.1)	85.5%
**Clinical department**				
Gynaecology-obstetric	11,472 (27.1)	23,157 (33.9)	20,741 (33.6)	89.6%
Surgical	4,983 (11.8)	11,285 (16.5)	8,739 (14.2)	77.4%
Paediatric	16,552 (39.2)	19,042 (27.8)	18,435 (29.9)	96.8%
Medical	9,221 (21.8)	14,842 (21.7)	13,716 (22.2)	92.4%
Not recorded	41 (0.1)	55 (0.1)	47 (0.1)	85.5%
**Blood products**				
Red blood cells	41,651 (98.5)	66,109 (96.7)	59,934 (97.2)	90.7%
Frozen fresh plasma	578 (1.4)	1,715 (2.5)	1,618 (2.6)	94.3%
Platelet concentrates	40 (0.1)	557 (0.8)	126 (0.2)	22.6%

## Data Availability

No data were used to support this study.

## References

[1] Somagari D. R., Sriram C. S., Rachamalla C. K. (2015). Haemovigilance study at a tertiary care hospital in the north-east of India. *ISBT Science Series*.

[2] Natukunda B., Schonewille H., Smit Sibinga C. T. (2010). Assessment of the clinical transfusion practice at a regional referral hospital in Uganda. *Transfusion Medicine*.

[3] Pélissier E., Nguyen L. (2000). Traçabilité des produits sanguins labiles: définition, réglementation, bilan et perspectives. *Transfusion Clinique et Biologique*.

[4] Ministère de la santé et de l'action humanitaire (1993). Loi no 93-5 du 4 janvier 1993 relative à la sécurité en matière de transfusion sanguine et de médicament. *Journal officiel français*.

[5] Ingrand P., Salmi L., Benz-Lemoine E., Dupuis M. (1998). Évaluation de la traçabilité effective des produits sanguins labiles à partir des dossiers médicaux. *Transfusion Clinique et Biologique*.

[6] Agence Nationale de Sécurité des Médicaments et des produits de santé. Rapport d’activité hémovigilance 2015. ANSM, 2016

[7] Tazi I., Loukhmas L., Benchemsi N. (2005). Hémovigilance : bilan 1995–2003 Casablanca. *Transfusion Clinique et Biologique*.

[8] Ouadghiri S., Atouf O., Brick C., Benseffaj N., Essakalli M. (2012). Traçabilité des produits sanguins labiles au Maroc : expérience de l’hôpital Ibn-Sina de Rabat entre 1999 et 2010. *Transfusion Clinique et Biologique*.

[9] Dahourou H., Tapko J., Nébié Y. (2012). Mise en place de l’hémovigilance en Afrique subsaharienne. *Transfusion Clinique et Biologique*.

[10] Dhingra N. http://wwwlive.who.int/entity/bloodsafety/makingsafebloodavailableinafricastatement.pdf.

[11] Dhingra N., Hafner V., Xueref S. (2003). Hemovigilance in Countries with Scarce Resources –A WHO Perspective. *Transfusion Alternatives in Transfusion Medicine*.

[12] Ministère de la santé, Burkina Faso. Décret n° 2012-1033/PRES/PM/MS/ du 28 décembre 2012 portant création, attribution et organisation d’un système national de vigilance des produits de santé à usage humain

[13] Ministère de la santé, Burkina Faso. Arrêté N°2014-589/MS du 9 juin 2014, portant directives nationales de Bonnes pratiques transfusionnelles au Burkina Faso

[14] Nébié K., Ouattara S., Sanou M. (2011). Poor procedures and quality control among nonaffiliated blood centers in Burkina Faso: An argument for expanding the reach of the national blood transfusion center. *Transfusion*.

[15] Sawadogo S., Kafando E., Nebie Y. K. Where are we with haemovigilance in Burkina Faso?.

[16] Décret N°2015-826/PRES-TRANS/PM du 13 juillet 2015 portant adoption du document de Stratégie Nationale de transfusion sanguine. JO Burkinabè N°41 du 08 OCTOBRE 2015

[17] de Vries R. R. (2009). Haemovigilance: recent achievements and developments in the near future. *ISBT Science Series*.

[18] Meza B. P. L., Lohrke B., Wilkinson R. (2014). Estimation of the prevalence and rate of acute transfusion reactions occurring in Windhoek, Namibia. *Blood Transfusion*.

[19] Ouadghiri S., Brick C., Benseffaj N., Atouf O., Essakalli M. (2017). Effets indésirables receveurs à l’hôpital Ibn Sina de Rabat : bilan 1999–2013. *Transfusion Clinique et Biologique*.

[20] Mahjoub S., Baccouche H., Raissi A., Ben Hamed L., Ben Romdhane N. (2017). Hémovigilance à Tunis (hôpital La Rabta) : bilan 2007–2013. *Transfusion Clinique et Biologique*.

[21] Mafirakureva N., Khoza S., Mvere D. A., Chitiyo M. E., Postma M. J., Van Hulst M. (2014). Incidence and pattern of 12 years of reported transfusion adverse events in Zimbabwe: A retrospective analysis. *Blood Transfusion*.

[22] South Africa National Blood Service. Haemovigilance Report 2012: South Africa National Blood Service; 2012

[23] Allain J.-P., Owusu-Ofori A. K., Assennato S. M., Marschner S., Goodrich R. P., Owusu-Ofori S. (2016). Effect of Plasmodium inactivation in whole blood on the incidence of blood transfusion-transmitted malaria in endemic regions: the African Investigation of the Mirasol System (AIMS) randomised controlled trial. *The Lancet*.

[24] Owusu-Ofori A. K., Owusu-Ofori S. P., Bates I. (2017). Detection of adverse events of transfusion in a teaching hospital in Ghana. *Transfusion Medicine*.

[25] Rouger P., Le Pennec P., Noizat-Pirenne F. (2000). Analyse des risques immunologiques en transfusion sanguine: Période 1991–1998. *Transfusion Clinique et Biologique*.

[26] Stainsby D. (2005). ABO incompatible transfusions—experience from the UK Serious Hazards of Transfusion (SHOT) scheme. *Transfusion Clinique et Biologique*.

[27] Vamvakas E. C., Blajchman M. A. (2009). Transfusion-related mortality: the ongoing risks of allogeneic blood transfusion and the available strategies for their prevention. *Blood*.

[28] Dujardin P.-P., Salmi L. R., Ingrand P. (2000). Errors in interpreting the pretransfusion bedside compatibility test. An experimental study. *Vox Sanguinis*.

[29] Dahourou H., Tapko J.-B., Kienou K., Nebie K., Sanou M. (2010). Recruitment of blood donors in Burkina Faso: how to avoid donations from family members?. *Biologicals*.

[30] Ouédraogo C. M. R., Ouédraogo A., Kaboré R. A. F. (2012). Analysis of blood transfusion requirements during the gravido-puerperal period in a hospital in Ouagadougou. *Field Actions Science Report*.

[31] Sawadogo S., Nebie K., Kafando E. (2016). Preparation of red cell concentrates in low-income countries: Efficacy of whole blood settling method by simple gravity in Burkina Faso. *International Journal of Blood Transfusion and Immunohematology*.

